# Injectable Cell-Laden Polysaccharide Hydrogels: In Vivo Evaluation of Cartilage Regeneration

**DOI:** 10.3390/polym14204292

**Published:** 2022-10-12

**Authors:** Yao Fu, Sanne K. Both, Jacqueline R. M. Plass, Pieter J. Dijkstra, Bram Zoetebier, Marcel Karperien

**Affiliations:** Department of Developmental BioEngineering, Faculty of Science and Technology, Tech Med Centre, University of Twente, P.O. Box 217, 7500 AE Enschede, The Netherlands

**Keywords:** injectable hydrogel, mesenchymal stem cells, chondrocytes, co-cultures, in vivo, subcutaneous implantation, cartilage regeneration

## Abstract

Previously, 5% *w*/*v* hyaluronic acid-tyramine (HA-TA) and dextran-tyramine (Dex-TA) enzymatically cross-linked hybrid hydrogels were demonstrated to provide a mechanically stable environment, maintain cell viability, and promote cartilaginous-specific matrix deposition in vitro. In this study, 5% *w*/*v* hybrid hydrogels were combined with human mesenchymal stem cells (hMSCs), bovine chondrocytes (bCHs), or a combination of both in a 4:1 ratio and subcutaneously implanted in the backs of male and female nude rats to assess the performance of cell-laden hydrogels in tissue formation. Subcutaneous implantation of these biomaterials showed signs of integration of the gels within the host tissue. Histological analysis showed residual fibrotic capsules four weeks after implantation. However, enhanced tissue invasion and some giant cell infiltration were observed in the HA-TA/Dex-TA hydrogels laden with either hMSCs or bCHs but not with the co-culture. Moreover, hMSC-bCH co-cultures showed beneficial interaction with the hydrogels, for instance, in enhanced cell proliferation and matrix deposition. In addition, we provide evidence that host gender has an impact on the performance of bCHs encapsulated in HA-TA/Dex-TA hydrogels. This study revealed that hydrogels laden with different types of cells result in distinct host responses. It can be concluded that 5% *w*/*v* hydrogels with a higher concentration of Dex-TA (≥50%) laden with bCH-hMSC co-cultures are adequate for injectable applications and in situ cell delivery in cartilage regeneration approaches.

## 1. Introduction

Articular cartilage injuries may occur as a result of either traumatic mechanical destruction or progressive mechanical degeneration. The combination of lack of blood supply and low mitotic activity of chondrocytes leads to limited ability to repair and regenerate articular cartilage [1,2,3,4]. Currently, injectable in-situ-formed hydrogels have emerged as promising cartilage tissue engineering strategies due to the ability to form three-dimensional, highly hydrated scaffolds after injection in aqueous form [5,6,7].

Injectable materials enable localized and straightforward delivery of cells and biomolecules via minimally invasive procedures without the associated risks of surgical implantation. These materials allow for the ability to fill irregular-shaped defects, avoiding the difficulty of prefabricating patient-specific defect shapes [8,9]. Moreover, hydrogels with different physical properties can also be designed and implanted in non-self-healing critical-size defects, temporarily replacing the extracellular matrix and assisting the healing process [10]. Previously, our group developed an injectable hybrid hydrogel composed of tyramine-conjugated hyaluronic acid (HA-TA) and dextran (Dex-TA) [11]. This hydrogel is formed in situ using a biocompatible enzymatic cross-linking reaction and supports survival of chondrocytes (CHs) and mesenchymal stem cells (MSCs) and tissue formation in vitro [12].

Tissue exposure to biomaterials triggers a foreign body response, a non-specific immune response process, which may result in persistent chronic inflammation and fibrotic encapsulation of the material [13,14,15]. In this inflamed environment, macrophages, lymphocytes, and their granular products contribute to the infiltration of foreign body giant cells (multi-nucleated fused macrophages) into the implantation site and the development of a collagen-rich fibrotic connective tissue layer surrounding the implant [14,16,17,18]. Degree of host response depends on the extent of homeostasis that is disturbed in the host by the injury, the implantation of the foreign material, and the properties of the material itself. Previously, an implant was considered biocompatible if it was encapsulated by an avascular layer of collagen without affecting its intended performance [18]. However, the impermeable nature of fibrous capsules, in some cases, results in poor mass transport and electrolyte diffusion between cell-laden implants and tissue, which impairs function, safety, and biocompatibility [19,20,21]. This is particularly relevant when these constructs are used in a tissue regeneration strategy.

In a previous study, we demonstrated that 5% *w*/*v* tyramine-conjugated hyaluronic acid and dextran (HA-TA/Dex-TA) hybrid hydrogels laden with bCHs provide a mechanically stable environment, maintain cell viability, and promote a cartilaginous-specific matrix deposition in vitro [12]. In the current study, 5% *w*/*v* hybrid hydrogels were combined with human mesenchymal stem cells (hMSCs) and bovine chondrocytes (bCHs) and then subcutaneously implanted in the backs of male and female nude rats for a four-week period. The cell laden hydrogels have a storage modulus (G’) of 1.9 kPa for pure Dex-TA, 3.2 kPa for 50/50 HA-TA/Dex-TA, and up to 9.6 kPa for pure HA-TA hydrogels [12]. The main objectives were to assess the response of cell-laden hydrogels with respect to tissue formation, characterize the reaction of neighboring tissues, and investigate the interaction between hybrid hydrogels and co-cultures or mono-cultures. Additionally, we investigated the effect of host gender differences on the outcomes of subcutaneous implantation of HA-TA/Dex-TA hydrogels laden with bCHs and hMSCs in the backs of male and female nude rats.

## 2. Materials and Methods

### 2.1. Materials

Dextran (40 kDa, pharmaceutical grade) was purchased from Pharmacosmos, Holbæk, Denmark. Sodium hyaluronate (27 kDa, pharmaceutical grade) was purchased from Contipro Pharma, Dolní Dobrouč, Czech Republic. Tyramine (99%), DMF (anhydrous, 99.8%), LiCl (99.0%), p-nitrophenyl chloroformate (PNC, 96%), pyridine (anhydrous, 99.8%), DMSO-d_6_ (99.9%), NaCl (≥99.0%), D_2_O (99.9 atom % D), horseradish peroxidase (HRP, 325 U/mg solid), and hydrogen peroxide (30%) were purchased from Sigma-Aldrich, Schnelldorf, Germany. Tyramine∙HCl salt (99%) was obtained from Acros Organics, Fair Lawn, NJ, USA. 4-(4,6-Dimethoxy-1,3,5-triazin-2-yl)-4-methyl-morpholinium chloride (DMTMM, 97%) was purchased from Fluorochem Ltd., Hadfield, UK. Ethanol (≥99.9%) and diethyl ether (≥99.7%) were purchased from Merck, Kenilworth, NJ, USA. Milli-Q water was used from the Milli-Q Advantage A10 system (Merck KGaA, Darmstadt, Germany) equipped with a 0.22 μm Millipak^®^ 40 Express filter.

### 2.2. Cell Culture and Expansion

bCHs were isolated from full-thickness cartilage knee biopsies from ~6-month-old calves according to the previously reported protocol [22]. bCHs were expanded in chondrocyte proliferation medium (Dulbecco’s modified Eagle’s medium (DMEM; Gibco, Billings, MT, USA) supplemented with 10% fetal bovine serum (FBS; Gibco), 0.2 mM ascorbic acid 2-phosphate (ASAP; Sigma, St. Louis, MO, USA), 0.4 mM proline (Sigma, St. Louis, MO, USA), 1× non-essential amino acids (Gibco), 100 U/mL penicillin, and 100 µg/mL streptomycin (Invitrogen, Carlsbad, CA, USA)).

Human-bone-marrow–derived MSCs were isolated as previously reported [23] and cultured in MSC proliferation medium (α-MEM (Gibco) supplemented with 10% FBS (Gibco), 1% L-glutamine (Gibco), 0.2 mM ASAP (Sigma), 100 U/mL penicillin, 100 µg/mL streptomycin (Invitrogen, Carlsbad, CA, USA), and 1 ng/mL bFGF)). The use of human material was approved by a local medical ethical committee. The medium was refreshed twice a week, and cells at Passage 3 were used for the experiments.

### 2.3. Synthesis of Polymers

Dextran-tyramine (Dex-TA) and hyaluronic acid-tyramine (HA-TA) were synthesized as previously reported [12]. Briefly, Dex-TA was synthesized by activation of dextran with PNC and subsequent aminolysis with tyramine adapted from Ramirez et al. [24]. HA-TA was prepared by amidation of the carboxyl groups of HA by tyramine, and the procedure was adapted from Rydergren et al. [25,26]. A detailed description of polymer synthesis can be found in the Supplementary Materials.

### 2.4. Hydrogel Formation

Hydrogel samples were prepared in a newly designed PTFE mold to produce identical hydrogels of 5 mm in diameter and 2.5 mm in height. After dissolving tyramine-conjugated polymers in sterile phosphate-buffered saline (PBS), polymer solutions were prepared and incubated with horseradish peroxidase (HRP, 40 U/mL) overnight at 4 °C. The mixture was then combined with different types of cells (hMSCs, bCHs, and bCH-hMSC co-cultures) in a concentration of 10 million cells/mL. For co-cultures, hMSCs and bCHs were mixed at a ratio of 80%/20% based on previous observations [27]. Freshly prepared 0.3% hydrogen peroxide (H_2_O_2_) was added to the mixture and immediately transferred to the mold using a 1 mL pipette after a brief vortex. The final concentrations of the gels were 5 % *w*/*v* polymer, 10 million cells/mL, 4 U/mL HRP, and 0.015% H_2_O_2_. HA-TA and Dex-TA were combined in 3 ratios (100:0, 50:50, and 0:100, represented by group HA, HA/Dex, and Dex, respectively). All conditions are listed in Table 1.

### 2.5. Hydrogel Implantation

**Figure 1:**
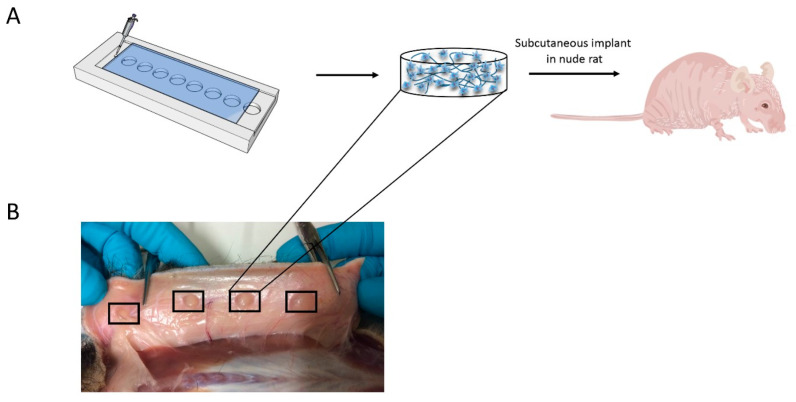
Experimental setup. (**A**) Schematic illustration of the outline of the experiment. Identical cell-laden hydrogels containing different types of cells were formed using a PTFE mold. Next, the prepared hydrogel constructs were implanted subcutaneously in the backs of 10 nude rats for 28 days. (**B**) Macroscopic observation of the different groups 28 days post-implantation. From left to right are representative pictures of groups HA-TA and co-culture, Dex-TA and bCHs, HA-TA/Dex-TA and hMSCs, and HA-TA/Dex-TA and bCHs, respectively. Frames denote the location of the implanted hydrogels.

After incubation in chondrogenic differentiation medium (DMEM supplemented with 0.2 mM ascorbic acid 2-phosphate (Sigma), 0.4 mM proline (Sigma), 100 U/mL penicillin, 100 µg/mL streptomycin (Invitrogen), 0.1 µM dexamethasone (Sigma), 100 µg/mL sodium pyruvate (Sigma), 50 µg/mL insulin-transferrin-selenite (ITS; Sigma), and 10 ng/mL transforming growth factor β-3 (TGF-β3; R&D Systems)) overnight, the hydrogel samples described above were implanted subcutaneously in the backs of 14-week old nude rats (Crl:NIH-Foxn1rnu) (Figure 1A). Each rat received carprofen (4 mg/kg) as an analgesic before the start of the procedure. Rats were induced with 4% isoflurane and maintained at 1.5–2% during the procedure. Skin was cleaned with 70% ethanol, and 4 subcutaneous pockets were created on each lateral side of the spine on the backs of 10 male rude rats (8 implants in total per rat). In each pocket, 1 hydrogel was inserted. Simultaneously, 2 samples (HA/Dex hydrogels laden with bCHs or hMSCs, respectively) were also implanted subcutaneously in the backs of another 10 female nude rats.

This experiment was randomized and approved by the local animal experimental committee. After 28 days, implants and respective surrounding tissue were harvested and fixed with 10% formalin and then incubated in cryomatrix (Thermo Scientific) overnight at 4 °C. Samples embedded in cryomatrix were snap-frozen using liquid nitrogen and stored at −80 °C.

### 2.6. Histology

Cryosections of 10 µm were cut using a cryotome (Thermo Shandon FSE, Thermo Fisher Scientific, Waltham, MA, USA)) and processed for histological evaluation with different staining methods. Hematoxylin-eosin (H&E) staining was performed using a standard protocol. Masson–Goldner Trichrome staining was performed to detect connective tissue and fibrous capsule thickness following the manufacturer’s instructions (Merck KGaA, Darmstadt, Germany). Briefly, samples were first placed in Weigert’s hematoxylin staining solution for 5 min and washed in tap water for 10 min. Sections were then shortly rinsed in 1% acetic acid, incubated in azophloxine solution for 10 min, then rinsed in 1% acetic acid again, followed by incubation in tungstophosphoric acid orange G solution for 1 min. Sections were again rinsed in 1% acetic acid for 30 s, followed by incubation in light green SF (Merck) for 2 min and another wash in 1% acetic acid. The thickness of the fibrous capsule and the inflammatory cell layer, i.e., a layer of cells mostly consisting of various immune cells, including mast cells, were measured for sections of all conditions. For each section, 4 points on each capsule around the implant were measured (*n* = 10). Peri-implant fibrotic capsule thickness was defined as the distance between the border of the fibrotic tissue adjacent to the implant and the muscle or fat tissue adjacent to the fibrotic capsule at the other end.

### 2.7. Naphthol AS-D Chloroacetate Esterase Staining

Naphthol AS-D staining was performed to stain granular cells such as neutrophils and mast cells. Chloroacetate esterase staining was performed following the manufacturer’s instructions (Sigma, St. Louis, MO, USA). Briefly, chloroacetate esterase staining solution was first prepared as described by the protocol. Cryosections were then incubated with the staining solution for 20 min at 37 °C in the dark. Sections were subsequently washed in distilled water and mounted with Faramount aqueous mounting medium (Dako, Glostrup, Denmark). All cells containing red granules were regarded as positive.

### 2.8. Immunohistochemistry Staining

For immunohistochemistry, endogenous peroxidase was blocked by incubating cryosections with 0.3% H_2_O_2_. After washing with PBS, sections were blocked in 5% bovine serum albumin to prevent non-specific binding. Slides were subsequently incubated overnight at 4 °C with a rabbit polyclonal antibody against COL II (Abcam, Cambridge, UK). Thereafter, sections were incubated with polyclonal goat anti-rabbit HRP-conjugated secondary antibody (Dako) for 30 min at room temperature, followed by development with the DAB Substrate Kit (Abcam). Counterstaining was performed with hematoxylin. Non-immune controls underwent the same procedure without primary antibody incubation.

### 2.9. Image Analysis and Statistical Analysis

All stained slides were scanned with the NanoZoomer 2.0-RS slide scanner (Hamamatsu, Sendai City, Japan). Stained sections were independently scored by three blinded individuals to assess tissue response. Fibrous capsule thickness and inflammatory cells were evaluated semi-quantitatively using NanoZoomer Digital Pathology software. Data are presented as mean ± standard deviation. Statistical significance was analyzed using one-way analysis of variance (ANOVA) with Tukey’s post-hoc analysis. A value of *p* < 0.05 was considered statistically significant.

## 3. Results

### 3.1. In Vivo Implantation in Nude Rats

Cell-laden hydrogel constructs were subcutaneously implanted in the backs of nude rats for 28 days to investigate cartilage matrix formation and tissue responses in vivo. The 28-day time point was chosen to focus on the inter-connections between cells and gels as well as co-cultured cells. Macroscopic observation of constructs, in all animals, showed proper integration with host tissue and no signs of edema or toxicity in the tissue surrounding the implants (Figure 1B). All hydrogel samples were clearly visible under the skin and maintained their structural integrity, indicating that they had not yet degraded significantly. In addition, no evident macroscopic inflammation of the tissue at the implantation site was observed.

**Figure 2:**
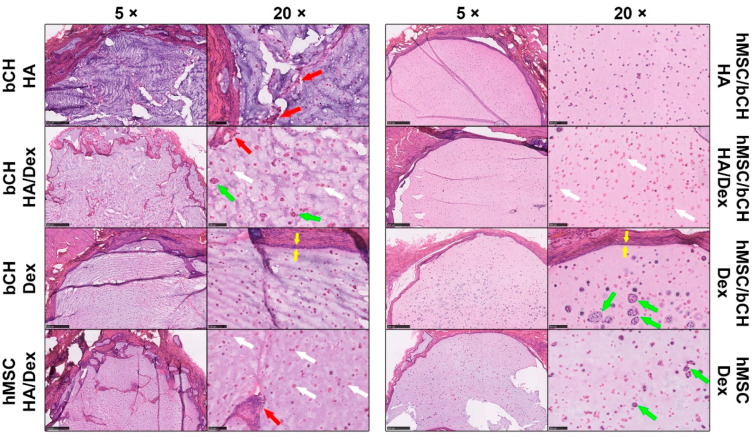
Histological analysis of in vivo response after 28 days of subcutaneous implantation. Representative histological sections of hydrogel samples stained with H&E. The left panel in each condition shows 5× magnification pictures (scale bars represent 500 µm), whereas the right panel shows 20× (scale bars represent 100 µm). The red arrows in the stained sections show giant cells, the green arrows show cell clusters, and the white arrows show the cells without visible nuclei, respectively. Yellow arrows at the material/tissue interface indicate a layer of inflammatory cells.

**Figure 3:**
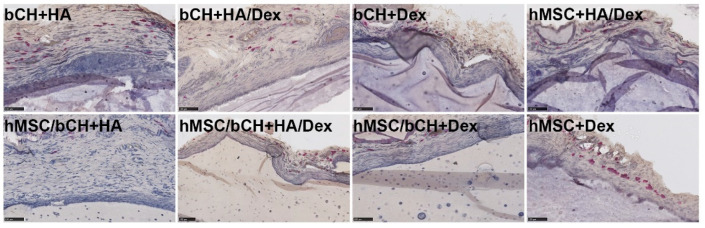
Neutrophil staining after 28 days of subcutaneous implantation. Representative figures of hydrogel samples with cells positively stained with chloroacetate esterase histochemistry. Positively stained cells displayed red granulation. Scale bars represent 100 µm.

**Figure 4:**
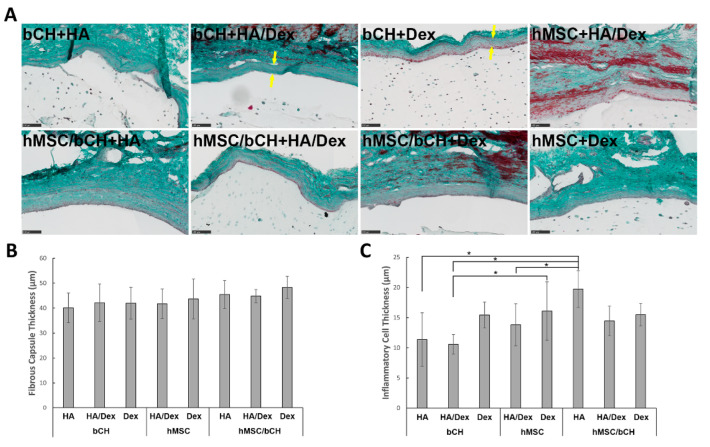
Histological characteristics of implant fibrous capsule after 28 days of subcutaneous implantation. (A) Representative Masson–Goldner trichrome staining of all hydrogels. All images show the interface between the host tissues (top of images) and the implant. As a result of the staining protocol, nuclei will be stained in dark brown to black, cytoplasm and muscles will appear brick red, the connective tissue will appear green, and erythrocytes will be bright orange. Yellow arrows in the stained sections indicate the fibrous capsule at the material/host interface. The thickness of the fibrous capsule (B) and the inflammatory cell layer (C), shown in Figure 2 with yellow arrows, were measured for sections of all conditions. Data are presented as mean with standard deviation as error bars for *n* = 10 biological replicates per hydrogel condition. Scale bars represent 100 µm. * represents *p* < 0.05.

To investigate the in vivo innate inflammatory response, the explanted samples, as well as the surrounding tissues, were histologically assessed (Figure 2, Figure 3 and Figure 4). Representative histological images of sections stained with hematoxylin and eosin (H&E) to examine the presence of tissue response are shown in Figure 2. Histological sections were evaluated via light microscopy and scored for tissue reaction, presence of giant cells, nuclei visibility, neutrophils, and cell clusters. The variable degrees of the inflammatory responses are summarized in Table 1. The presence of the above inflammatory responses was scored from absence (−) to profound presence (+++). The stained sections show that the HA hydrogels with encapsulated bCHs do not display a solid gel, but a more porous structure, which could be caused by the degradation of the gel. Mixing in Dex-TA progressively decreased the structure’s porosity. Remarkably, it was not present in the HA hydrogels laden with a mixture of hMSCs and bCHs.

A new interface consisting of inflammatory cells (e.g., macrophages) was generated between the hydrogels and host tissues after implantation, but no acute inflammatory reaction was observed in all types of samples. Stained figures show that most of the explanted constructs displayed smooth edges at the material–tissue interface. Hydrogel samples containing only bCHs or hMSCs showed a more abundant chronic tissue reaction, especially in the cell-laden HA-TA/Dex-TA constructs. Enhanced tissue invasion and some giant cell infiltration were observed in the HA-TA/Dex-TA hydrogels either laden with bCHs or hMSCs. However, tissue reaction was barely noticeable in co-culture-laden hydrogels irrespective of the hydrogel composition.

Regarding cell morphology, similar results were found in most of the implanted samples. Encapsulated cells showed a round morphology and homogeneous distribution throughout the constructs. Meanwhile, proliferating cells were found in the conditions of HA/Dex with bCHs, Dex with hMSCs, and Dex with co-cultures, which were present as newly generated cell clusters. In particular, in the Dex-encapsulated co-cultures, enhanced newly formed cell clusters were observed. Of interest, cellularity analysis showed that in HA/Dex construct nuclei visibility significantly decreased compared to other conditions. Most of the cells in the HA/Dex samples were stained pink, so no nuclei were visible, regardless of the cell type encapsulated.

Next, we checked for the presence of neutrophils surrounding the implantation site. Neutrophil granulocytes represent the abundant cell type in peripheral blood and normally arrive as the first immune cells at an implant site and disappear in the course of the following days. Chloroacetate esterase histochemistry is a well-known method to detect granular cells such as neutrophils and mast cells. Positively stained granular cells, which were mostly identified as mast cells, were only sporadically present in the gel-tissue boundary and seldom into the implant. These results demonstrate the absence of significant inflammation processes (Figure 3).

Trichromatic Masson–Goldner staining is most suitable to depict the structure of connective tissues and cells and to assess the fibrous capsule formation by collagen deposition. Representative Masson–Goldner trichrome staining of the cell-laden hydrogel samples at 28 days post-implantation is shown in Figure 4A. This staining revealed that all samples were surrounded by a fibrous capsule. Additionally, a semi-quantitative assessment of capsule thickness and inflammatory cells at the implant surface is shown in Figure 4B,C. The average thickness of the peri-implant capsule in all conditions ranged from 40 µm to 48 µm, and no significant differences were observed between the different hydrogels (Figure 4B). A layer of inflammatory cells was present at the material–tissue interface (Figure 2). The layer thickness was highest around HA hydrogels with co-cultures compared to others (Figure 4C).

### 3.2. Co-Culture-Laden Hydrogels Present Positively Deposition of Cartilage Matrix

**Figure 5:**
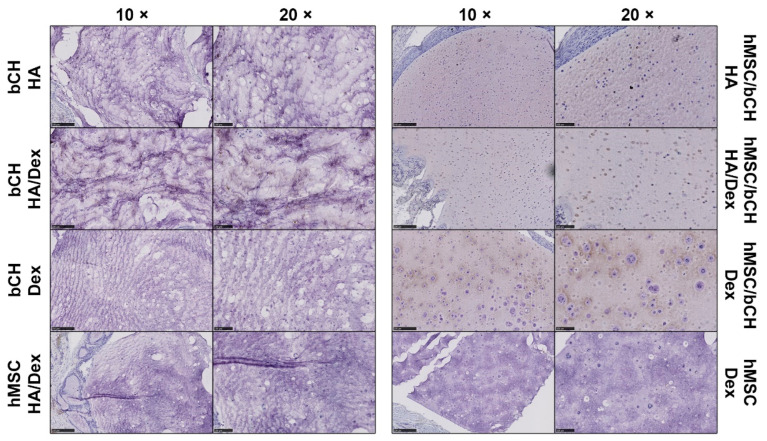
Matrix deposition after 28 days of subcutaneous implantation. Representative immunohistochemical staining of the hydrogel samples for COL II. Positive protein expression stained in dark brown. The left panel in each condition shows 10× magnification pictures (scale bars represent 250 µm), whereas the right panel shows 20× (scale bars represent 100 µm).

Immunohistochemistry was performed to investigate the expression of proteins that indicate cartilage matrix production. Collagen type II, which is the primary type of collagen present in articular cartilage, was chosen in this study. We rarely observed the expression of COL II in the constructs laden with hMSCs after subcutaneous implantation (Figure 5). We did observe some pericellular staining for COL II within the hydrogels loaded with bCHs. Positively stained protein expression was more apparent in co-culture-laden hydrogels. Notably, intense deposition of COL II was observed in Dex-TA hydrogels laden with co-cultures. Moreover, the histochemical analysis also revealed that the cartilage matrix formation was more dominant at the periphery of the hydrogels. These results indicate that the co-culture system and Dex-TA hydrogel could effectively promote the appropriate interactions or stimulations for chondrogenesis, leading to the facilitated secretion of chondrogenic extracellular matrix and cartilaginous tissue formation during in vivo subcutaneous implantation.

### 3.3. Gender Difference Shows Impact on the Performance of Chondrocyte Laden Hydrogels

**Figure 6:**
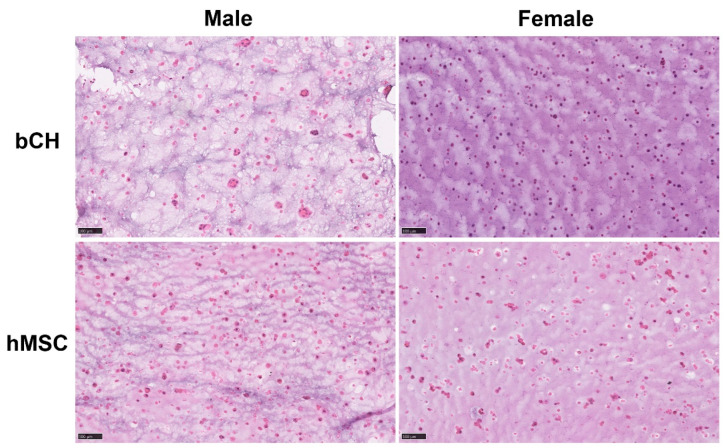
Histological analysis comparing implants laden with bCHs or hMSCs in either male (left) or female (right) nude rats. 5% *w*/*v* HA/Dex hydrogels laden with hMSCs or bCHs were implanted subcutaneously into the backs of both male and female rude rats. Representative histological sections of hydrogel samples stained with H&E. Scale bars represent 100 µm.

To investigate the impact of gender differences on the outcomes, we implanted HA/Dex hydrogels laden with bCHs or hMSCs, respectively, subcutaneously in the backs of another 10 female nude rats. The histological analysis results are summarized in Table 2. Surprisingly, we found gender differences in the performance of implanted hydrogels; however, this impact was mainly observed for hydrogels laden with bCHs but not with hMSCs. Irrespective of gender, all the samples formed a clear thin fibrous capsule surrounding the hydrogels, while no sign of acute inflammatory response was observed. In the hMSC-laden samples, most of the assessed markers were present similarly in samples from both males and females. However, as shown in Figure 6, the outcomes from bCH-laden hydrogels clearly show gender differences. Compared to explant samples from female rats, the in vivo environment in male rats increased the size of encapsulated bCHs, depressed the nuclei visibility of encapsulated bCHs, and promoted the formation of cell clusters.

## 4. Discussion

In previous research, we developed and characterized an injectable hydrogel system to be applied in cartilage tissue regeneration [12]. In such hydrogels, cells can be homogeneously distributed and in vitro evaluation showed high biocompatibility. We utilized different compositions of hyaluronic acid and dextran hybrid hydrogels and demonstrated that 5% *w*/*v* hydrogels showed, after preoperative incubation in chondrogenic differentiation medium, enhanced deposition of cartilage matrix. Cartilage matrix deposition significantly increases mechanical properties. In this work, the safety and biocompatibility of these hybrid hydrogels laden with bCHs and/or hMSCs were tested by subcutaneous implantation in the backs of nude rats for four weeks. This study demonstrated that, as a major result, the hydrogels present limited immune response and formation of a small fibrotic capsule surrounding the material. As a second major aspect, bCHs-hMSCs co-cultures show beneficial interaction with the biomaterials, for instance, in enhanced cell proliferation and matrix deposition. Finally, this research revealed that hydrogels with these types of cells resulted in distinct tissue responses, which indicated the possibility of personalized regeneration approaches based on the situation of individual patients. Of interest, gender appeared to influence the performance of bCH-laden hydrogels after subcutaneous implantation.

The utilization of injectable hydrogels in cartilage regeneration is considered a promising strategy due to the minimally invasive procedure [8,28]. In situ gelation enables the formed hydrogel to quickly set its volume, adapt to the shape of the defect, and establish an efficient integration with the host tissue [9,29]. Moreover, this system can deliver cells and/or bioactive agents of interest in a non-harsh way and keep them at the implantation site [30]. However, implantation of biomaterials triggers a series of host responses at the injury site, including material/tissue interactions, acute and chronic inflammation, granulation tissue development, foreign body reaction, and fibrous capsule development [17,18]. Accordingly, this body response to the foreign material compromises the in vivo functionality and durability of the implanted material [14]. To evaluate the response of a host to hte first contact with this biomaterial, 5% *w*/*v* hybrid hydrogels laden with bCHs, hMSCs, or co-cultures thereof were subcutaneously implanted in the backs of nude rats, and histological analysis was conducted to assess the inflammatory processes associated with the implantation, as well as the integration with the host tissue.

After four weeks, all implanted samples showed proper integration with host tissue and no signs of granulation tissue development or toxicity in the tissue surrounding the implants. Histological analysis revealed that all hydrogel conditions elicited no acute inflammatory response. H&E staining of histological sections also revealed a homogeneous distribution of the cells within the matrix, with the cells exhibiting the common round-shaped phenotype characteristic. While no signs of acute inflammation response could be detected, a thin (40–48 µm) fibrotic capsule, as an indication of chronic inflammation, was observed around all the implants. Mostly mast cells, but not neutrophils, were sporadically present in the gel-tissue boundary and seldom into the implant. However, no polymorphonuclear and mono-nuclear cells were visible, and no significant differences were observed between the different hydrogel conditions. These results demonstrate the absence of significant inflammation processes. The in vivo performance of these hydrogels, along with previous data, suggests that HA, Dex, and hybrid hydrogels are suitable for injectable applications in tissue regeneration approaches with good in vivo safety and biocompatibility.

However, it should be noticed that some differences in foreign body reaction to the hydrogel matrix were observed after four weeks. For instance, hydrogels laden with either bCHs or hMSCs induced some tissue reaction, particularly in the hydrogels of HA-TA only. Moreover, the foreign body giant cells, a typical feature of the foreign body reaction, were only observed in somewhat larger numbers in mono-culture cell-laden hydrogels prepared from HA-TA. In this case, the encapsulation of hMSCs did not elicit the formation of foreign body giant cells, as previously reported [31,32].

Nevertheless, Dex-TA hydrogels laden with either bCHs or hMSCs showed moderate tissue reaction in the matrix and less giant cell formation. Moreover, hydrogels with HA-TA showed a porous structure when laden with either bCHs or hMSCs. This can be explained by the degradation of HA in vivo. HA is a major component of the cartilage extracellular matrix and exhibits rapid degradation behaviors in vivo due to its high water-absorbing properties and enzymatic degradation [33]. The space left after HA degradation promotes the surrounding tissue invasion and may explain the infiltration of foreign body giant cells. Moreover, HA-TA/Dex-TA constructs show significantly depressed nuclei visibility compared to other conditions. Most of the cells in HA-TA/Dex-TA gels were stained pink, while nuclei were barely visible, regardless of the cell type encapsulated. We hypothesize that the in vivo environment in the male rats probably increased the size of encapsulated cells in HA/Dex constructs, which impacts the sectioning and in turn affects the staining results.

Interestingly, all these performances were attenuated in the co-culture environment. No tissue reaction and giant cell formation were observed in hydrogels laden with both bCHs and hMSCs. Previous studies demonstrated the beneficial interactions between the cells in bCH-hMSC co-cultures [34]. The in vivo performance indicated that this beneficial interaction also affects the performance of hydrogels compared to hydrogels laden with either hMSCs or bCHs. The underlying mechanism and potential role of this interplay between cells and biomaterials need further investigation.

Moreover, enhanced newly formed cell clusters were observed in Dex-TA-encapsulated bCH-hMSC co-cultures, which indicated that Dex-TA hydrogels provide the environment to support the beneficial interactions in bCH-hMSC co-cultures. Moreover, only hydrogels laden with co-cultures present deposition of cartilage matrix. Notably, coherent with the in vitro study, intense deposition of type II collagen was observed in pure Dex-TA hydrogels with co-cultures. In conclusion, together with results from previous studies, HA-TA/Dex-TA hydrogels with a high concentration of Dex-TA (≥50%) provide the opportunity to create optimal biomaterials for cartilage tissue regeneration, while bCH-hMSC co-cultures stimulate interaction with these hydrogels. These data suggest that further in situ study is needed for the development of a fully functional cartilage tissue-engineered construct that can be applied clinically. It should be noted though that the subcutaneous implantation site may have influenced the outcome of cartilage matrix production. It seems feasible that orthotopic implantation may facilitate cartilage matrix production over the subcutaneous implantation site. Additionally, different combinations of hydrogel and cells showed distinct differences in mechanical properties, degradation, and chondro-induction features. These properties are important considerations in the design of precision biomaterials to enable the survival, differentiation, and transplantation of biomaterial-cell-based combination approaches. With the growing interest in personalized therapeutic approaches [35,36], combination therapies have vital potential for their ability to sense and respond to the therapeutic needs of individual patients. The different outcomes of the in vivo performance in this work highlight the potential application of personalized regeneration based on the situation of individual patients.

Animal models are essential to assess the value of current and future tissue engineering therapies, which play a critical role in many domains of study in medicine and biology [37,38]. Multiple factors need to be considered in selecting an appropriate animal model, such as animal size, age, gender, economic cost, ethical concerns, and potential for clinic transition [38,39]. In this work, we investigated the impact of gender differences on our injectable hydrogels for cartilage tissue engineering. The histological assessment indicated that the gender of the host has an effect on the performance of the implanted hydrogels. However, the impact is mainly on the hydrogels laden with bCHs, not with hMSCs. Due to the space limitation in this study, we only chose cell-laden HA/Dex hydrogels. Further studies need to proceed on the animals with normal immune systems and other hybrid hydrogels to check if there is any outcome change.

In this work, the evaluation of the in vivo response upon subcutaneous implantation of hyaluronic acid and dextran hybrid hydrogels was conducted in nude rats, revealing proper integration with the surrounding tissues and the presence of a residual fibrotic capsule. Moreover, the in vivo performance revealed the interaction of bCH-hMSC co-cultures with biomaterials, suggesting their further study towards the development of functional cartilage tissue-engineered constructs for personalized application. Taken together, the results from this work, along with previous data, show that 5% *w*/*v* Dex-TA hydrogels laden with bCH-hMSC co-cultures provide an adequate support matrix for chondro-induction between hMSC and bCH co-cultures, stimulating cartilage matrix formation.

## Figures and Tables

**Table 1:** Histological scoring of the in vivo sections.

Conditions		Tissue Reaction	Giant Cells	Nuclei Visibility	Neutrophil	Cell Cluster
bCHS	HA	+±	+	++±	±	−
	HA/Dex	++	+	+	−	+
	Dex	+	±	++±	−	−
hMSCS	HA/Dex	++	+±	+	±	−
	Dex	+	+	++	±	+
bCH/hMSC co-cultures	HA	−	−	+++	−	−
	HA/Dex	−	±	+	−	−
	Dex	−	±	++	±	++

Twenty-eight days after subcutaneous implantation in male nude rats (*n* = 10 rats in each group), sections were assessed via various markers to evaluate tissue response. The presence of the above inflammatory components was scored from absence (−) to profound presence (+++).

**Table 2:** Histological analysis of the in vivo sections from different genders.

Samples	Gender	Tissue Reaction	Giant Cells	Nuclei Visibility	Cell Size (µm)	Collagen Capsule	Capsule Thickness (µm)	Neutrophil	Cell Cluster
bCHS + HA/Dex	M	++	+	+	17.8	+	40.7	−	+
	F	+	+	++±	12.2	+	44.8	−	−
hMSCs + HA/Dex	M	++	+±	+	17.7	+	42.5	±	−
	F	+±	+	+	17.4	+	45.2	±	−

Twenty-eight days after subcutaneous implantation in nude rats of different genders (*n* = 10 rats in each group), sections were assessed via various markers to evaluate the impact of gender difference on the performance of the cell-laden hydrogels. M represents male, and F represents female.

## Data Availability

Not applicable.

## Supplementary Materials

The following supporting information can be downloaded at: https://www.mdpi.com/article/10.3390/polymers1933438/s1, Detailed synthetic protocols for Dex-TA and HA-TA.
