# Supplementary material for: Injectable Cell-Laden Polysaccharide Hydrogels: In Vivo Evaluation of Cartilage Regeneration
Source: Polymers (Basel). 2022 Oct 12;14(20):4292. doi: 10.3390/polym14204292 (PMC9607606; doi:10.3390/polym14204292)
Supplement: Supplementary file 1 [file polymers-14-04292-s001.zip › polymers-1933438-supplementary.pdf]

polymers

MDPI

Supplementary Materials

Injectable Cell-Laden Polysaccharide Hydrogels: In Vivo Evaluation of Cartilage Regeneration

Yao Fu [†,‡], Sanne Both, Jacqueline Plass, Pieter J. Dijkstra, Bram Zoetebier and Marcel Karperien *

Supplementary Materials

Synthesis of Dextran-p-nitrophenyl Carbonate

LiCl (4.0 g, dried at 115 °C) and dextran (5.00 g, 30.8 mmol r.u.) are weighed into a 500 mL three necked round bottom flask equipped with a stirrer bar. The flask is evacuated and refilled with nitrogen 3 times, after which it is left under vacuum at 95 °C for 1.5h. After thoroughly drying, the flask was filled with nitrogen and 200 mL of anhydrous DMF was added via a cannula while stirring. The flask was then equipped with a thermometer and the mixture was heated to 95 °C while stirring the solution. Once the dextran was completely dissolved, the solution was cooled to 0 °C and anhydrous pyridine (2.0 ml, 25.8 mmol) was added. Subsequently, freshly sublimed para-nitrophenyl chloroformate (2.5 g, 12.4 mmol) was added in small portions, while keeping the temperature below 2 °C. After 1 hour, the reaction mixture was poured into 1 L of ice-cold ethanol. The precipitate was filtered off (Por 4) and washed with cold ethanol (3 × 100 mL) and subsequently with diethyl ether (3 × 100 mL). After drying under vacuum, the product was obtained as a white powder (6.00 g, 30.7 mmol r.u., 99 % yield, DS20%). 1H-NMR (400 MHz, DMSO-d6): δ(ppm) = 3.0–4.0 (saccharide ring protons, m, 6H); 4.2–5.8 (anomeric and hydroxyl protons, m, 4H); 7.58 (Ar o-CH, d, 2H); 8.34 (Ar m-CH, d, 2H).

Synthesis of Dextran-Tyramine

Dextran-PNC (6.00 g, 30.7 mmol r.u., 6.15 mmol p-nitrophenyl carbonate) was weighed into a 250 mL three necked round bottom flask equipped with a stirrer bar. The flask was evacuated and refilled with nitrogen 3 times, after which the flask was filled with nitrogen and 100 mL of anhydrous DMF was added via a cannula while stirring. Once the dextran was completely dissolved, tyramine (1.69 g, 12.3 mmol) was added. After 1 hour, the reaction mixture was poured into 1 L of ice-cold ethanol. The precipitate was filtered off (Por 4) and washed with cold ethanol (3 × 100 mL) and subsequently with diethyl ether (3 × 100 mL). After drying under vacuum, the crude product was obtained as a white powder. The crude product was dissolved in 75 mL of Milli-Q water and dialyzed against Milli-Q water for 3 days (MWCO 3500 Da), followed by filter sterilization and freeze-drying yielding the product as a white foam (5.04 g, 28.0 mmol, 92 % yield, DS10%). 1H-NMR (400 MHz, DMSO-d6): δ(ppm) = 3.0–4.0 (saccharide ring protons, m, 6H); 4.2–5.8 (anomeric and hydroxyl protons, m, 4H); 6.67 (Ar m-CH, d, 2H); 6.99 (Ar o-CH, d, 2H).

The calculation of the DS of dextran-TA and dextran-PNC is based on the integrals of 4.2–5.8 ppm (corresponding to the 4 anomeric protons from dextran), compared with the integral of the aromatic protons of tyramine (6.60–6.75 and 6.90–7.07) or para-nitrophenyl (7.40–7.65 and 8.20–8.40). The DS of dextran is given as the percentage of saccharide units modified in dextran.

Synthesis of Hyaluronic Acid-Tyramine

Sodium hyaluronate (5.00 g, 12.5 mmol r.u.) was dissolved in 500 mL Milli-Q water in a 1 L round bottom flask equipped with a stirrer bar. While stirring at room temperature, 4-(4,6-dimethoxy-1,3,5-triazin-2-yl)-4-methylmorpholinium chloride (DMTMM, 3.46

g, 12.5 mmol, 1eq) and tyramine hydrochloride (TA·HCl, 2.17 g, 12.5 mmol, 1eq) were added subsequently. The addition of DMTMM and TA·HCl was repeated after 24 and 48 hours. After 72 hours, 40 mL NaCl (sat) was added to the reaction mixture and the reaction mixture was poured into 2.5 L cold ethanol. The crude product was isolated by centrifugation at 5000 rpm followed by drying in vacuo. The crude product was dissolved in 75 mL Milli-Q water and dialyzed against Milli-Q water for 3 days (MWCO 1000 Da). Filter sterilization and lyophilization yielded the product as a white foam (5.10 g, 12.4 mmol, 99 % yield, DS10%). 1H-NMR (400 MHz, D2O): δ(ppm) = 1.98 (acetyl-CH3, s, 3H); 2.75 (2-CH2, s, 2H); 2.90 (1-CH2, s, 2H); 3.2–4.2 (saccharide ring, m, 10H); 4.34 (s, 1H); 4.43 (d, 1H); 6.84 (Ar m-CH, d, 2H); 7.16 (Ar o-CH, d, 2H).

The degree of substitution (DS) was calculated based on the integral of the methyl group at 1.98 ppm, compared to the integral of the tyramine signals at 6.80–6.87 and 7.10–7.21 ppm. The DS of hyaluronic acid is given as the percentage of COOH groups modified in hyaluronic acid (i.e. per disaccharide).
